# Evaluation of sedimentation rate methodology reveals an unusual pediatric subpopulation with lupus or lupus-like syndrome and hemolytic anemia

**DOI:** 10.1186/1546-0096-10-S1-A122

**Published:** 2012-07-13

**Authors:** Edward C Wong, Deborah Yuan, Claas Hinze, Lawrence Jung

**Affiliations:** 1 Children's National Medical Center, Washington, DC, USA

## Purpose

Sedimentaion rate is often used to manage pediatric patients with rheumatologic disease. Most management decisions are dependent on studies which have used Wintrobe or Westergren sedimentation rate methodologies. However, these methods suffer from the need for relatively large amounts of blood and long turn-around times. Determination of sedimentation rate using laser kinetic rate determination has allowed calibration to Westergren methods, low volume of blood needed for testing and very rapid results. We sought to compare the Wintrobe method to the ESR Stat method (kinetic method; HemaTechnologies, Lebanon NJ) to determine suitability of the ESR Stat method for patient testing.

## Methods

We performed a prospective comparison between the traditional Wintrobe and ESR Stat sedimentation rates in consecutive pediatric patients at a tertiary care pediatric hospital. Wintrobe and ESR Stat sedimentation data was fitted using a logarithmic model. Outliers were defined as those samples with ESR Stat sedimentation rates greater than 80 mm/hr and Wintrobe sedimentation rates less than 30 mm/hr (normal or mildly elevated sedimentation rate). Retrospective chart review was performed on all patients undergoing testing.

## Results

A total of 131 pediatric patients (with one patient undergoing repeat testing because of sedimentation rate discrepancy) were tested. Age range was 18 months to 34 years with 29% being male. A logarithmic model appeared to best fit the data (R^2^ = 0.7768) and is seen below. Of interest was the identification of four patients who had apparently normal or mildly elevated sedimentation rates by the Wintrobe method versus an extremely high (greater than 120 mm/hr) by the ESR Stat method. These patients were noted to have lupus or lupus-like syndrome and a history of hemolytic anemia. Non-outlier samples were from patients who did not this combination of disease morbidities.

**Figure 1:**
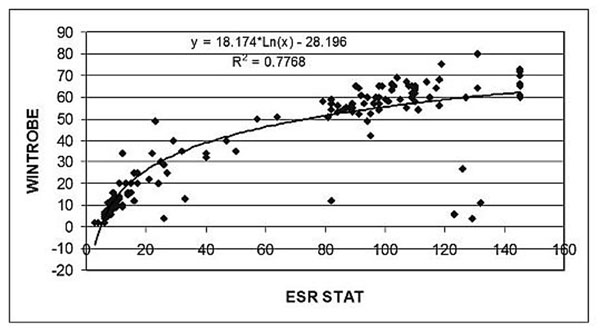


## Conclusion

Discrepancies in Wintrobe and ESR Stat sedimentation rates may identify a subgroup of with lupus (or lupus-like syndrome) and a history of hemolytic anemia. Careful consideration of methodology is needed when sedimentation rate testing is performed on pediatric lupus patients.

## Disclosure

Edward C. Wong: None; Deborah Yuan: None; Claas Hinze: None; Lawrence Jung: None.

